# The use of V02 and VC02 to optimise respiratory support and to wean mechanically ventilated patients after major abdominal surgery

**DOI:** 10.1186/2197-425X-3-S1-A315

**Published:** 2015-10-01

**Authors:** MV Petrova, SD Beeharry, AV Butrov, R Mohan, OL Bessolitsina

**Affiliations:** PFUR, Moscow, Russian Federation

**Keywords:** Indirect calorimeter, V02 and VC02, Mechanical ventilation, Abdominal surgery

## Introduction

The incidence of patients requiring ICU admission is as high as 32-55% after major abdominal surgeries due to severe abdominal complications. Mechanical ventilation is sometimes necessary during intensive treatment. Prognosis remains poor with mortality rate of 29-42%. Success in weaning from mechanical ventilation in these patients remains a big problem.

## Objectives

To use V02 and VC02 (indirect calory parameters) to optimise respiratory support and to wean patients from mechanical ventilation.

## Methods

26 patients after major abdominal surgery requiring Mechanical ventilation (11 women and 15 men with an average age of 52.4 ± 9.2) were randomly selected, from mid of 2014 to 2015. Patients were on mechanical ventilation for a at least 5 days. Engstrom Carestation **Indirect Calorimeter´s gas analyser** (E-COVX) was used to measure the changes in oxygen consumption (V02) carbon dioxide production (VC02) on different modes of mechanical ventilation with stepwise reduction of mandatory breaths in Bi-level mode (from 12 to 6) with Pressure support of 16 mm H_2_O, followed by continuous positive airway pressure (CPAP/PSV) mode with decreasing Pressure support from 16 to 6 mm H_2_O was used as weaning method. Cardiac output was also measured using bio-impedance method. Weaning procedure lasted 20-24 hours with changes in parameters noted every 2-3 hrs.

## Results

The V02 and VC02 of patients in Bi-level mode were of 255 ± 38 ml/min and 188 ± 32 ml/min respectively. In weaned patients, a slight increase in ΔV02 of 23 ± 4 ml/min (9.1%) and significant increase in ΔVC02 of 31 ± 5 ml/min (16.5%) occurred gradually with the decrease in pressure support. An insignificant increase in cardiac output from 3.6 ± 0.4 l/min to 3.9 ± 0.4 l/min was noted. 16 out of 26 patients were successfully weaned but in 6 of them mechanical ventilation had to be restarted. 10 patients could not be weaned at all and their ΔV02 was of 77 ± 10 ml/min (30.1%) and ΔVC02 was of 54 ± 7 ml/min (28.7%). These changes was also related with significant decrease in cardiac output to 3.1 ± 0.3 l/min.

## Conclusions

Changes in ΔV02 and ΔVC02 parameters during weaning may be used as an alternate to Arterial blood gas analysis as they respond quicker to respiratory and metabolic distress. Thus, patients ventilator parameters could be altered accordingly in time without any significant respiratory and cardiac decompensation.

